# Lung‐ and liver‐dominant phenotypes of Korean eight constitution medicine have different profiles of genotype associated with each organ function

**DOI:** 10.14814/phy2.15459

**Published:** 2022-09-06

**Authors:** Changkeun Kim, Kyung‐Won Hong, Da‐Hyun Park, Sukyung Chun, Sooyeon Oh, Youngji Park, KyongChol Kim, Sang‐Woon Choi, Heejin Jo

**Affiliations:** ^1^ Chaum Life Center CHA University Seoul Republic of Korea; ^2^ John Eight Constitution Medical Clinic Seoul Republic of Korea; ^3^ Theragen Bio Co. Ltd. Suwon‐si Republic of Korea; ^4^ WellCare Clinic Seoul Republic of Korea; ^5^ School of Public Health and Health Sciences University of Massachusetts Amherst Massachusetts USA

**Keywords:** eight constitution medicine (ECM), genome‐wide association study (GWAS), Korean medicine, phylogenetic analysis, single nucleotide polymorphism (SNP)

## Abstract

Eight Constitution Medicine (ECM), a ramification of traditional Korean medicine, has categorized people into eight constitutions. The main criteria of classification are inherited differences or predominance in the functions of organs, such as the liver or lung, diagnosed through ECM‐specific pulse patterns. This study investigated the association between single nucleotide polymorphism (SNP) genotypes and ECM phenotypes and explored candidate genetic makeups responsible for each constitution using a genome‐wide association study (GWAS). Sixty‐three healthy volunteers, who were either categorized as the Hepatonia (HEP, *n* = 32) or Pulmotonia (PUL, *n* = 31) constitution, were enrolled. HEP and PUL are two contrasting ECM types representing the dominant liver and lung phenotypes, respectively. SNPs were analyzed from the oral mucosa DNA using a commercially available microarray chip that can identify 820,000 SNPs. We conducted GWAS using logistic regression analysis and additive mode genotypes and constructed phylogenetic trees using the SNPhylo program with 8 SNPs specific for the liver phenotype and 15 SNPs for the lung phenotype. Although genome‐wide significant SNPs were not found, the phylogenetic tree showed a clear difference between the two constitutions. This is the first observation suggesting genetic involvement in the ECM and can be extended to all ECM constitutions.

## Graphical abstract

HEP and PUL are two contrasting ECM types representing the dominant liver and lung phenotypes, respectively. We conducted GWAS using logistic regression analysis and additive mode genotypes, and constructed phylogenetic trees using the SNPhylo program with 8 SNPs specific for the liver phenotype and 15 SNPs for the lung phenotype. Although genome‐wide significant SNPs were not found, the phylogenetic tree showed a clear difference between the two constitutions. This is the first observation suggesting genetic involvement in the ECM and can be extended to all ECM constitutions.

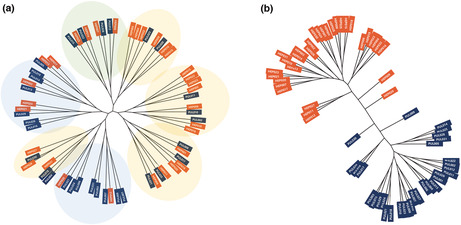

## 1 INTRODUCTION

The concept that the treatment of a pathological condition needs to be different from person to person has been a common strategy in traditional Korean medicine before the introduction of personalized medicine in Western medicine. Traditional Korean medicine practitioners have tried to provide individually tailored treatments such as acupuncture, moxibustion or herbal medicines for better results, and to do so, they categorized humans based on a group of specific characteristics, called constitution. Eight Constitution Medicine (ECM), a constitutional medical typology in Korean medicine, was proposed by Do‐won Kuon, a doctor of traditional Korean medicine (Kuon & Berhardt, 2011), in 1965 (Kuon, 1965). It classifies individuals into eight constitutions—Pulmotonia (PUL), Colonotonia (COL), Renotonia (REN), Vesicotonia (VES), Pancreotonia (PAN), Gastrotonia (GAS), Hepatonia (HEP), and Cholecystonia (CHO) (Kuon, 2003)—based on the inherited functional strength of individual organs such as lung, colon, kidney, bladder, pancreas, stomach, liver, and gall bladder (Kim, Jang, et al., 2020; Kuon, 2003).

As individuals in a different constitution have different pathophysiologic and psychologic characteristics, they need to receive acupuncture treatment consisting of different acupoint combinations (Kuon, 1965; Kuon & Berhardt, 2011), and be informed of a different preventative approach, such as detailed dietary recommendations and susceptibility to constitution‐specific vulnerable diseases (Cho et al., 2013; Kim, Kuon, et al., 2020; Kuon, 2003; Kuon & Berhardt, 2011). The eight constitutions are diagnosed mainly by the radial arterial pulse (Kuon, 1974), which is one of the most important phenotypic traits of the ECM; clinical experience of the practitioner is therefore critical for the consistency and accuracy of ECM pulse diagnosis (Shin et al., 2009). To improve classification, alternative diagnostic tools have been developed. A questionnaire that asks for other outstanding phenotypes (such as subjective physiologic characteristics including sweating tendency, digestive function, and response to specific food) is available, but it needs further improvement for practical use (Lee et al., 2012). In this regard, we questioned whether the phenotypes of ECM that are considered inherited conditions can be evaluated by a certain genotype. Genotyping can be helpful for the precise classification and can result in a better understanding of the genetic background of ECM.

Single nucleotide polymorphisms (SNPs) are the most common types of genetic variability. As SNPs are responsible for over 80% of the variations between two people (Marth et al., 2001), they have been extensively studied to investigate correlations between genotype and phenotype. Genome‐wide association study (GWAS) is a common approach to study variations in unrelated individuals (Dehghan, 2018; McCarthy et al., 2008). Among many SNP analyses, a phylogenetic tree was developed for evolutionary studies and has demonstrated the association between or within organisms based on genetic data. The SNPhylo program is a newly designed pipeline to construct a phylogenetic tree from large SNP data and allow researchers to interpret their data graphically (and more rapidly) by showing a distinctive difference between species (Lee et al., 2014).

In this pilot study, we selected two opposite traits out of the eight constitutions, HEP and PUL, and investigated their SNPs with the hypothesis that there would be informative genetic variants that could differentiate between HEP and PUL. GWAS and phylogenetic analysis were conducted to identify genetic makeups that could determine the genetic difference between HEP and PUL.

## 2 MATERIALS AND METHODS

### 2.1 Subjects

**FIGURE 1:**
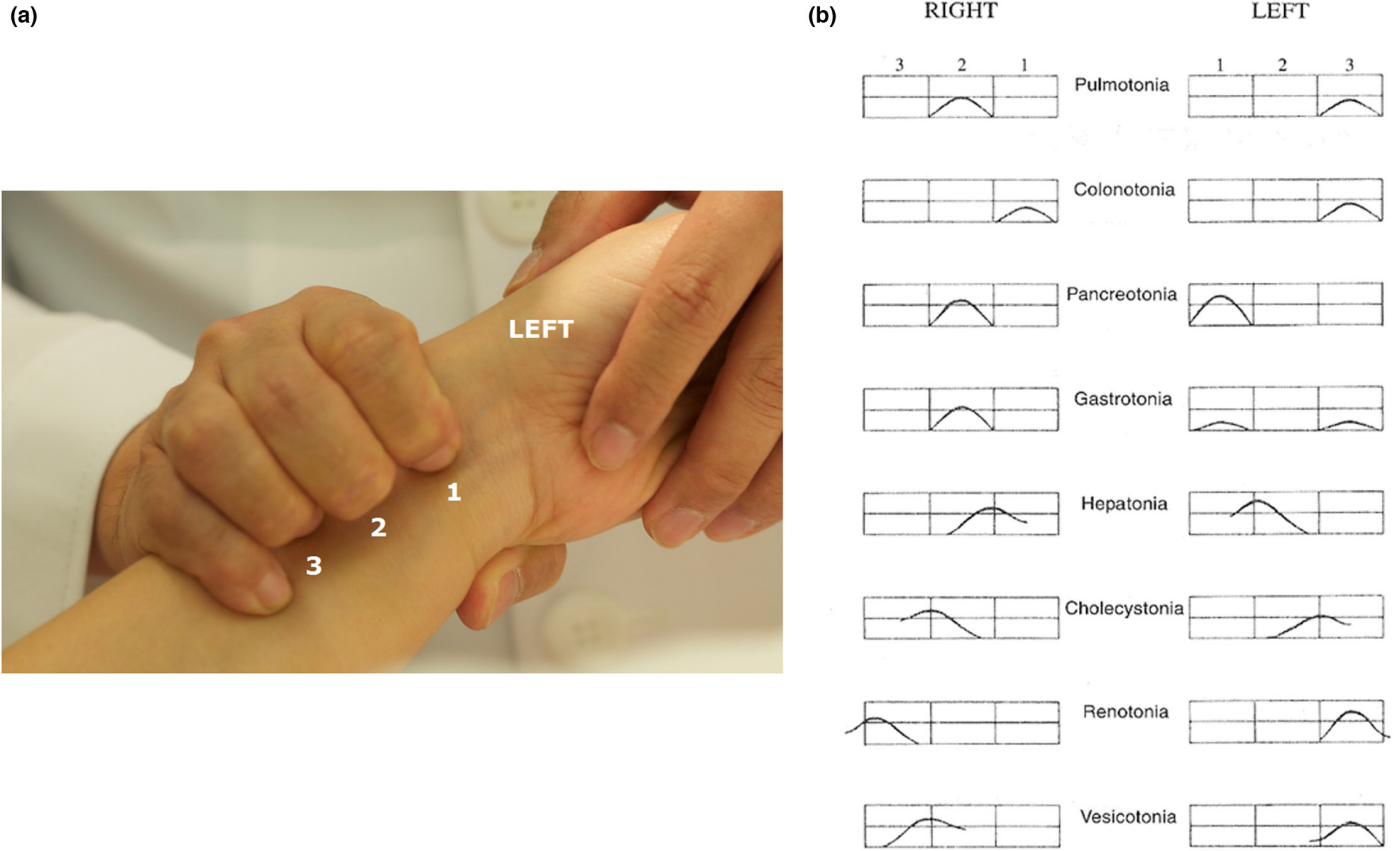
Eight pulse patterns of the radial artery in eight constitutional medicine (a) ECM pulse diagnosis is different from the traditional Korean pulse diagnosis, in terms of the location at which it is performed and the method that is used. The diagnostic procedure is as follows: First, the physician asks the patient to lie down and grips the patient's LEFT wrist (LEFT) with the doctor's right three fingers contacting the patient's radial artery. The position of the physician's index finger (1), middle finger (2) and ring finger (3) is placed on the radial artery line that is 5cm below the patient’ s radial styloid process. Next, the physician presses the patient's radial artery until the pulse can no longer be felt. Then, the physician releases the pressure of three fingers applied to the artery and catches where the first strongest pulse bounces up. The same process is also performed on the patient's right wrist with the physician's left hand (RIGHT). The physician should take into consideration the inclination of the patient's radius and give the three fingers the same strength to press the patient's wrist evenly. (b) The diagnosis of eight constitutions were made using unique pulse patterns composed of the combination of pulse formations on both the left and right radial arteries. The curved line in the present figure indicates the first pulse wave increase; the box indicates the pulse strength.

This study was conducted with healthy volunteers aged >20 years enrolled at Chaum Life Center, Seoul, Korea, from April 2019 to July 2019. We included the two most opposing constitutions in ECM, HEP and PUL, in this study to determine whether genetic difference can be found between the two constitutions. Volunteers' constitutions were diagnosed using the pulse diagnostic method proposed by Dr. Kuon as shown in Figure 1. The diagnoses were made twice to ensure accuracy and reproducibility by one skillful ECM specialist who has been experienced in ECM for about 20 years. Exclusion criteria included chronic pathological conditions such as cancers, hypertension, cardiovascular diseases, diabetes mellitus, psychiatric disorders, or any health problems that may significantly modify their phenotypes. Cases where the pulse diagnostic method was unavailable such as the vascular malformation of the radial artery or unilateral upper‐limb loss, were also excluded. A total of 63 subjects were enrolled in this study. All recruitment and conduct of the study were approved by the Institutional Review Board of CHA Bundang Medical Center, CHA University (CHAMC 2019–03‐006).

### 2.2 SNP microarray and genotyping analysis

Genomic DNA was extracted from the oral mucosa scrapings using a Theragen DNA collection kit (Theragen Bio). The DNA samples of 31 HEP and 32 PUL were analyzed with the Theragen Precision Medicine Research Array (PMRA) chip (Thermo Fisher Scientific), a customized Asian PRMA array consisting of more than 820,000 reliable SNPs. The SNPs were further filtered for quality using PLINK v.1.90 software (Chang et al., 2015) under the following criteria: genotype call rates (<0.97), minor allele frequency (MAF) (<0.05), genotype missing rate (<0.05), individual call rate (<0.1), and Hardy–Weinberg equilibrium (<0.05). The genome‐wide association study (GWAS) was conducted using HEP and PUL constitutions coded 2 and 1, respectively with controlling for sex as the covariate. Logistic regression analysis and additive mode genotypes were used, and the Manhattan plots of the GWAS signals were drawn up using the “ggplot2” package (Wickham, 2009) in R version 3.6.2 (R Core Team, 2019). We investigated the top SNPs for the significant index of the HEP and PUL constitutions at *p* < 1 × 10^−3^. We also search for more SNPs associated with the liver or lung phenotypes previously reported by the GWAS catalog (Buniello et al., 2019), as HEP and PUL constitutions are defined with the inherited functional strength of the liver and lung in ECM.

### 2.3 Phylogenetic analysis

To explore the genomic differences between HEP and PUL groups, we constructed a phylogenetic tree using SNPhylo software (https://github.com/thlee/SNPhylo) (Lee et al., 2014). The SNPhylo software was used for phylogenetic analysis based on the SNP data. We conducted phylogenetic analysis with the maximum likelihood algorithm and 1000 bootstraps. Among the genome‐wide chip SNPs, we constructed phylogenetic trees in two ways; first, we used all the genome‐wide SNPs with *p* < 0.05 in GWAS results; second, we selected the SNPs with *p* < 1 × 10^−3^ and the SNPs previously reported to be related to liver function, which is known to be the strongest in HEP, or lung function, which is known to be the strongest in PUL, among the GWAS results.

## 3 RESULTS

**FIGURE 2:**
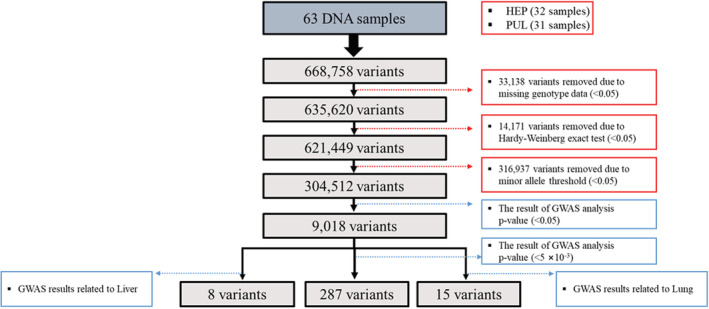
The schematic flowchart of SNP selection for Korean ECM (32 HEP group and 31 PUL group)

All participants (*n* = 63) in this study were Korean, consisting of 19 men and 44 women (16 men and 16 women in the HEP group; 3 men and 28 women in the PUL group). The median age was 55 years, ranged from 24 to 87 years. The age and sex distributions of the subjects in HEP and PUL group are shown in the Table S1. There was no significant difference noted between HEP and PUL group in age but in sex. All subjects from both groups were healthy individuals with no diagnosable diseases. All DNA samples extracted from the oral mucous membrane showed high dish QC (DQC >0.82) and high call rates (CR >0.97). A total of 304,512 SNPs were selected for GWAS (Figure 2) among 668,758 SNPs successfully genotyped with the Precision Medicine Research Array (PRMA) chip after removing SNPs with a genotype call rate of less than 0.97 and an individual call rate of less than 0.1. A total of 33,138 SNPs with genotypes missing rate <0 0.05, 14,171 SNPs with a Hardy–Weinberg equilibrium (HWE) test where *p* < 0.05, and 316,937 SNPs with minor allele frequency (MAF) < 0.05 (PLINK v1.90) were removed.

### 3.1 Genome‐wide association analysis

**FIGURE 3:**
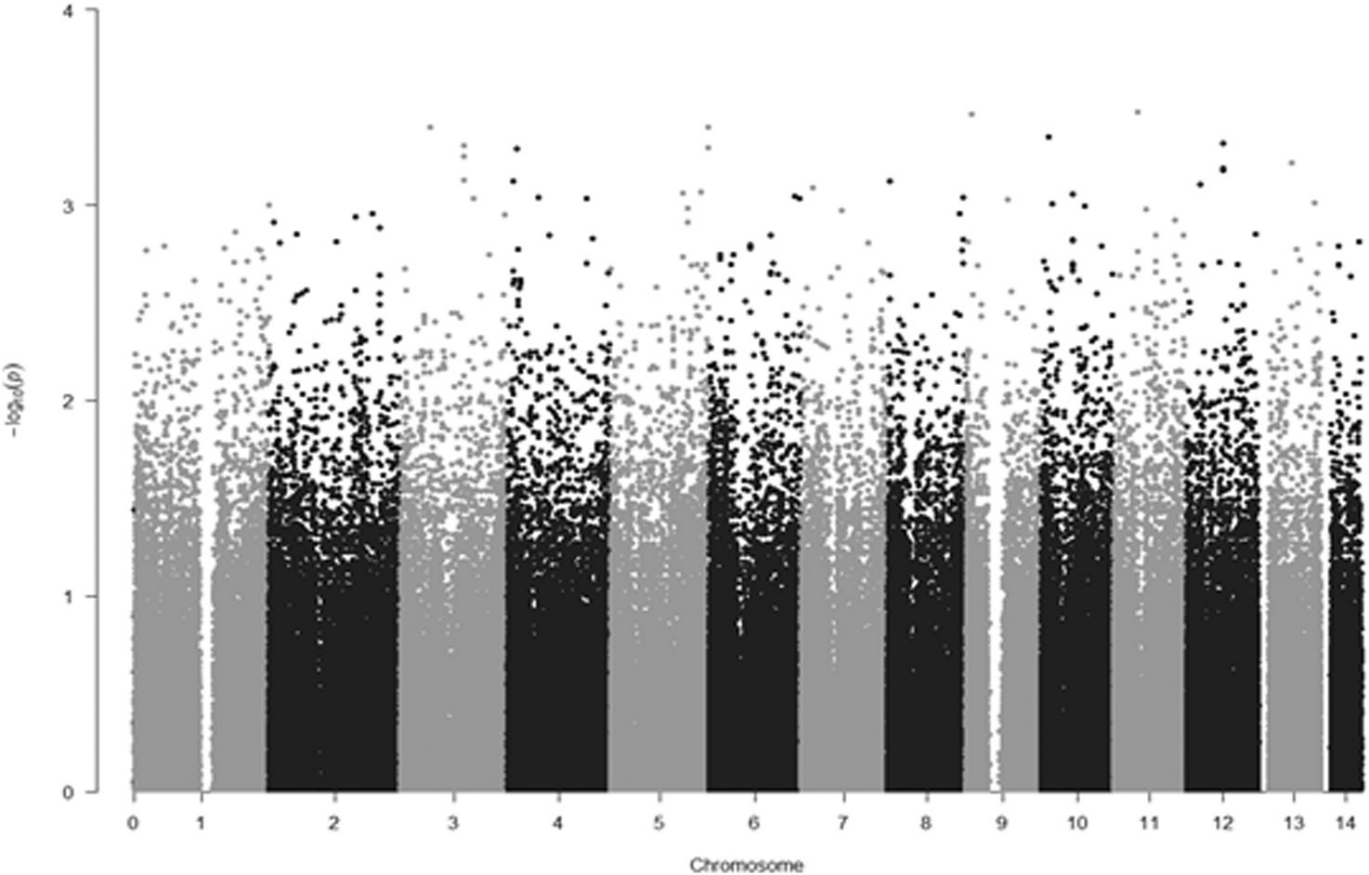
Manhattan plot of the GWAS results for ‘Hepatonia’ (HEP) and ‘Pulmotonia’ 2 (PUL) constitutions; − log10 (*p*‐value) of alleles for HEP against PUL were plotted against 3 chromosomal position. All the *p*‐values are over 5 × 10–5.

The genome‐wide distribution of *p*‐values across all chromosomes for HEP and PUL is shown in the Manhattan plot (Figure 3), suggesting that there were no genome‐wide significant SNPs (*p* < 5 × 10^−8^) and no suggestive SNPs (*p* < 1 × 10^−5^). We listed up the SNPs at *p* < 1 × 10^−3^ in HEP and PUL groups (Table 1). Although the level of significance of these SNPs was not high, the results indicated that the top 32 SNPs could be considered as the significant index of HEP and PUL constitutions and may have a moderate association or tendency with those two constitutions in ECM.

**TABLE 1:** SNPs associated with ‘Hepatonia’ (HEP) and ‘Pulmotonia’ (PUL) constitutions at *p* < 1E‐03

CHR	SNP	BP	A1	A2	MAF	OR^a^	L95	U95	P	Feature	Gene
11	rs7946090	44,592,908	A	G	0.287	6.744	2.377	19.13	3.34.E‐04	intron_variant	CD82
9	rs10816235	9,920,242	G	T	0.2726	0.1481	0.05204	0.4214	3.45.E‐04	intron_variant	PTPRD
3	rs13058921	54,137,943	G	T	0.244	0.1473	0.05099	0.4254	4.01.E‐04	intron_variant	CACNA2D3
5	rs248184	179,275,279	C	A	0.2323	0.1473	0.05099	0.4254	4.01.E‐04	intron_variant	ADAMTS2
10	rs4748151	15,279,060	T	C	0.3947	0.2487	0.1143	0.5411	4.50.E‐04	intron_variant	FAM171A1
12	rs2306393	68,314,981	C	T	0.4745	6.589	2.285	19	4.84.E‐04	missense_variant	MDM1
3	rs62269264	116,670,814	A	G	0.3869	5.72	2.143	15.26	4.97.E‐04	intergenic_region	LSAMP‐TUSC7
5	AX‐35245709	180,069,422	T	C		9.318	2.648	32.79	5.07.E‐04	intron_variant	RNF130
4	rs2102702	17,024,068	A	C	0.2787	0.131	0.04161	0.4125	5.15.E‐04	intergenic_region	LDB2‐LOC101929123
4	rs1514983	17,024,831	A	G	0.2732	0.131	0.04161	0.4125	5.15.E‐04	intergenic_region	LDB2‐LOC101929123
3	AX‐247301836	116,710,060	A	C		0.2158	0.09029	0.5159	5.64.E‐04	intron_variant	TUSC7
13	rs7326543	61,402,698	G	A	0.392	5.592	2.09	14.96	6.08.E‐04	intergenic_region	MIR3169‐PCDH20
12	rs2870812	68,325,436	G	A	0.4721	6.33	2.192	18.28	6.49.E‐04	splice_region_variant	MDM1
12	rs962976	68,326,847	G	A	0.4722	6.287	2.181	18.12	6.64.E‐04	missense_variant	MDM1
3	rs10934365	116,725,899	T	C	0.3192	0.2191	0.0907	0.5295	7.45.E‐04	intergenic_region	TUSC7‐MIR4447
3	rs1432364	116,726,477	T	G	0.3191	0.2191	0.0907	0.5295	7.45.E‐04	intergenic_region	TUSC7‐MIR4447
4	rs12508896	10,544,229	G	A	0.4448	0.25	0.1116	0.5603	7.60.E‐04	intron_variant	CLNK
8	rs1154099	4,503,122	G	A		0.25	0.1116	0.5603	7.60.E‐04	intron_variant	CSMD1
12	rs1602185	25,421,712	A	G	0.2646	0.1694	0.0601	0.4776	7.87.E‐04	intergenic_region	KRAS‐LMNTD1
7	rs2539967	20,937,592	C	T	0.4525	0.2138	0.08667	0.5276	8.15.E‐04	intron_variant	LINC01162
5	rs1864975	167,460,624	C	T	0.2625	0.1668	0.0582	0.4778	8.53.E‐04	intron_variant	TENM2
5	rs329120	134,526,066	T	C	0.4109	5.52	2.02	15.08	8.64.E‐04	upstream_gene_variant	JADE2
10	rs1649081	58,532,684	G	G	0.4966	3.821	1.735	8.416	8.78.E‐04	intron_variant	BICC1
6	rs901363	158,757,313	C	T	0.1853	8.5	2.401	30.09	9.06.E‐04	missense_variant	SYTL3
8	rs9886648	139,861,686	T	C	0.2286	0.164	0.05633	0.4773	9.11.E‐04	intron_variant	TRAPPC9
4	rs17087289	56,955,516	C	T	0.2593	0.1556	0.05179	0.4672	9.13.E‐04	intergenic_region	REST‐NOA1
3	rs1540734	135,557,947	A	C	0.4446	4.776	1.894	12.04	9.23.E‐04	intergenic_region	EPHB1‐PPP2R3A
6	rs28499100	167,477,040	A	G	0.2491	6.077	2.089	17.68	9.28.E‐04	upstream_gene_variant	LOC105378127
4	AX‐41378891	146,617,731	A	G		5.133	1.949	13.52	9.29.E‐04	intergenic_region	RNU1‐44P‐POU4F2
9	rs963469	76,112,335	A	C	0.4351	0.2742	0.1274	0.5901	9.37.E‐04	intron_variant	PCSK5
13	rs1412951	103,588,302	G	A	0.43	0.2083	0.08199	0.5294	9.79.E‐04	intergenic_region	LINC01309‐DAOA‐AS1
10	rs11012537	21,126,655	G	A	0.228	0.1949	0.07371	0.5154	9.81.E‐04	intron_variant	C10orf113

Abbreviations: A1, Minor frequency allele; A2, Major frequency allele; BP, Base‐pair position; CHR, Chromosome; L95, Lower 95% confidence interval; OR, Odds ratio; P, unadjusted *p*‐value; SNP, single nucleotide polymorphism; U95, Upper 95% confidence interval.

^a^ The odds ratio (OR) > 1.0 indicates the individuals with minor frequency allele (A1) would be more likely to be classified under the PUL constitution than those with major frequency allele homozygotes.

Considering the odds ratio (OR) in Table 1, among the top 32 significant SNPs associated with HEP and PUL constitutions at *p* < 1 × 10^−3^, 19 SNPs (rs10816235, rs13058921, rs248184, rs4748151, rs2102702, rs1514983, AX‐247301836, rs10934365, rs1432364, rs12508896, rs1154099, rs1602185, rs2539967, rs1864975, rs9886648, rs17087289, rs963469, rs1412951, and rs11012537) whose OR is at <1 might be considered more likely to constitute HEP. The other 13 SNPs (rs7946090, rs2306393, rs62269264, AX‐35245709, rs7326543, rs2870812, rs962976, rs329120, rs1649081, rs901363, rs1540734, rs28499100, and AX‐41378891) could be considered to indicate a more possible association with the PUL constitution.

### 3.2 Constitution‐specific phenotype‐related SNPs

HEP and PUL constitutions are quite different ECM constitutions in that their inherited strengths in the liver and lung functions are opposite in order. Therefore, we searched for SNPs that are known to be associated with the liver and lung phenotypes using GWAS catalog (Buniello et al., 2019). And 8 SNPs for the liver phenotypes (Table 2) and 15 SNPs for the lung phenotypes (Table 3) were selected. Considering the OR in Table 2 and Table 3, 5 SNPs (rs2499604, rs222054, rs12743824, rs4949718, and rs7820212) associated with the liver phenotype and 9 SNPs (rs10436951, rs3009947, rs12201912, rs546131, rs2571445, rs541601, rs10113175, rs9299346, and rs755249) associated with the lung phenotype, whose OR is at <1, might be thought to constitute HEP constitution. And 3 SNPs (rs251891, rs12145922, and rs13030978) associated with the liver phenotype and 6 SNPs (rs2348418, rs764129, rs2637254, rs2608029, rs10824305 and rs2579762) associated with the lung phenotype, whose OR is at >1, could be thought to constitute PUL constitution. However, all those 23 SNPs are at *p* < 0.05 and did not pass the significance level commonly used in GWAS studies (*p* < 5 × 10^−8^).

**TABLE 2:** GWAS results associated with the liver

CHR	SNP	BP	A1	OR^a^	L95	U95	P	Trait
1	rs2499604	237,940,201	C	0.2654	0.1151	0.6119	1.85E‐03	Non‐alcoholic fatty liver disease histology(AST)
4	rs222054	71,738,582	G	0.2437	0.08958	0.6628	5.68E‐03	Non‐alcoholic fatty liver disease histology(other)
1	rs12743824	99,317,401	C	0.3868	0.1731	0.8643	2.06E‐02	Non‐alcoholic fatty liver disease histology(other)
1	rs4949718	76,433,779	T	0.3715	0.1542	0.8952	2.73E‐02	Liver enzyme levels (aspartate transaminase)
5	rs251891	115,714,665	T	2.385	1.075	5.291	3.24E‐02	Liver injury in combined anti‐retroviraland
1	rs12145922	88,680,551	A	2.666	1.042	6.821	4.08E‐02	Liver enzyme levels (gamma‐glutamyl transferase)
8	rs7820212	52,506,268	T	0.1048	0.01201	0.9142	4.12E‐02	Immunoglobulin light chain (AL) amyloidosis(liver)
2	rs13030978	191,252,512	T	2.225	1.003	4.938	4.92E‐02	Liver enzyme levels (gamma‐glutamyl transferase)

Abbreviations: A1, Minor frequency allele; BP, Base‐pair position; CHR, Chromosome; L95, Lower 95% confidence interval; OR, Odds ratio; P, unadjusted *p*‐value; SNP, single nucleotide polymorphism; U95, Upper 95% confidence interval.

^a^ The odds ratio >1.0 indicates the individuals with minor frequency allele (A1) would be more likely to PUL constitution than those with major frequency allele homozygotes.

**TABLE 3:** GWAS results associated with the lung

CHR	SNP	BP	A1	OR^a^	L95	U95	P	Trait
1	rs10436951	243,199,378	G	0.2135	0.07486	0.609	3.89E‐03	Lung cancer in ever smokers
1	rs3009947	218,515,813	C	0.3052	0.1153	0.8082	1.69E‐02	Lung function (FEV1/FVC)
6	rs12201912	116,908,643	A	0.3374	0.1352	0.8421	1.99E‐02	Lung function (FEV1/FVC)
12	rs2348418	28,536,581	C	2.459	1.119	5.405	2.52E‐02	Lung function (FVC)
3	rs764129	185,830,129	T	2.237	1.071	4.673	3.22E‐02	Lung function (FEV1/FVC)
10	rs2637254	76,552,244	A	2.497	1.08	5.772	3.23E‐02	Lung function (FVC)
8	rs2608029	128,157,880	C	4.422	1.129	17.32	3.28E‐02	Lung adenocarcinoma
11	rs546131	34,830,213	G	0.3422	0.1269	0.9228	3.41E‐02	Lung disease severity in cystic fibrosis
10	rs10824305	75,380,711	C	2.492	1.063	5.844	3.57E‐02	Lung function (FEV1/FVC)
2	rs2571445	217,818,431	A	0.4545	0.2152	0.9599	3.87E‐02	Lung function (FVC)
11	rs541601	126,139,605	T	0.4544	0.2092	0.9868	4.62E‐02	Lung function (FEV1/FVC)
8	rs10113175	127,057,790	C	0.288	0.08398	0.9876	4.77E‐02	Lung cancer in never smokers
9	rs9299346	101,611,505	A	0.4361	0.1908	0.9966	4.91E‐02	Methotrexate‐induced interstitial lung disease in rheumatoid arthritis
10	rs2579762	76,559,121	C	2.348	1.001	5.507	4.98E‐02	Lung function (FEV1/FVC)
1	rs755249	39,529,402	T	0.2444	0.05982	0.9989	4.98E‐02	Lung function (FEV1/FVC)

Abbreviations: A1, Minor frequency allele, BP, Base‐pair position; CHR, Chromosome; L95, Lower 95% confidence interval; OR, Odds ratio; SNP, single nucleotide polymorphism; U95, Upper 95% confidence interval; P, unadjusted *p*‐value.

^a^ The odds ratio >1.0 indicates the individuals with minor frequency allele (A1) would be more likely to PUL constitution than those with major frequency allele homozygotes.

### 3.3 Construction of a phylogenetic tree with HEP and PUL constitution‐specific SNPs

**FIGURE 4:**
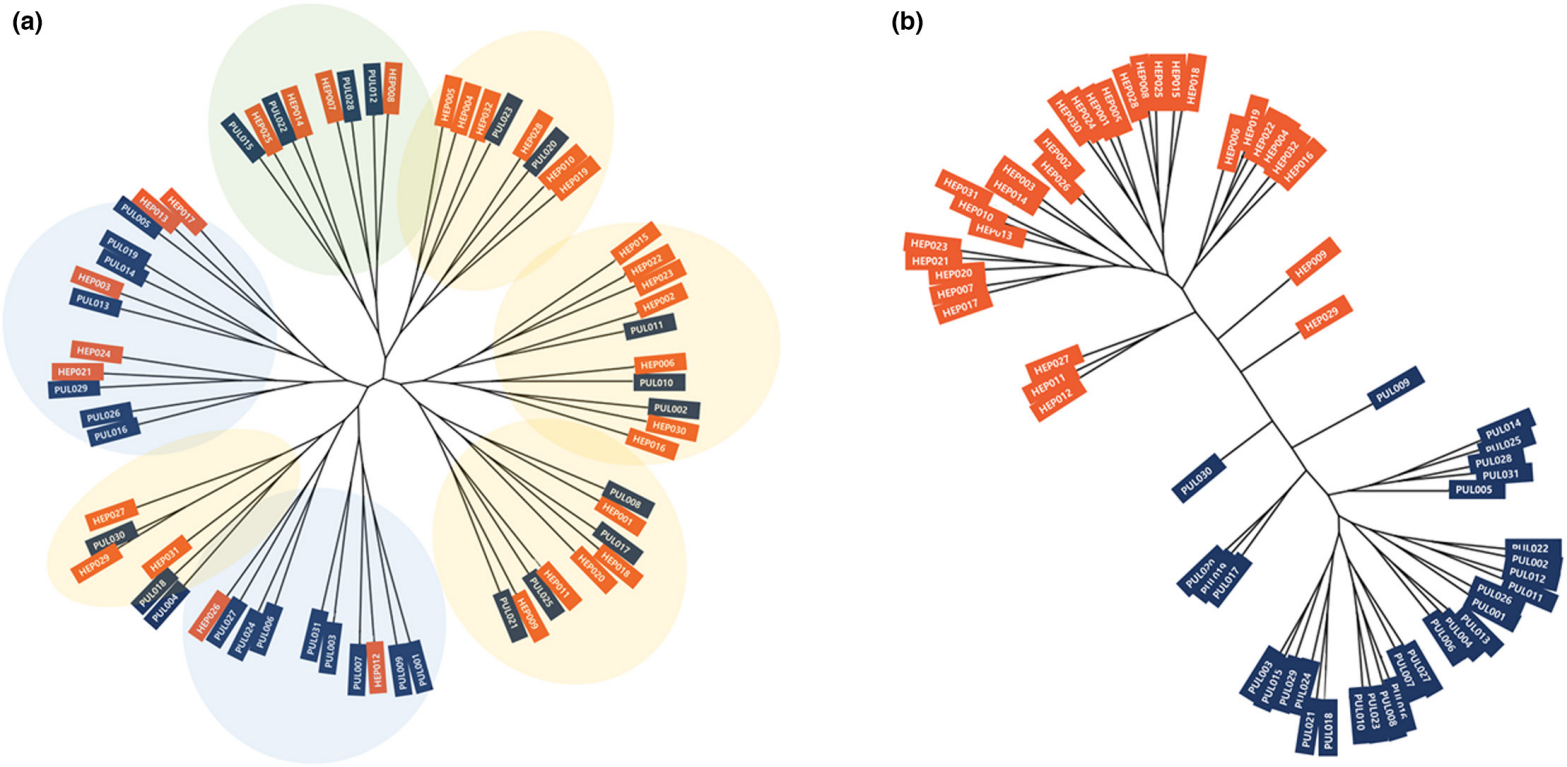
Constitutional phylogenetic trees showing HEP and PUL (a) maximum likelihood tree (bootstrap 100) was constructed by the SNPhylo using all the genome‐wide SNPs with GWAS *p*‐value <0.05. The seven clusters in the tree did not show a distinct difference between HEP and PUL constitutions. (b) the same genome‐wide phylogenetic analysis was conducted by the SNPhylo using 8 SNPs related to the liver phenotype, 15 SNPs related to the lung phenotype selected from GWAS results on HEP and PUL subjects and 287 SNPs with GWAS *p*‐value <5 × 10–3. This lung and liver phenotype‐related SNP phylogenetic tree showed two clusters reflecting a distinct difference between HEP and PUL. HEP, Hepatonia; PUL, Pulmotonia.

Phylogenetic analysis was conducted using SNPhylo to investigate the genomic properties of HEP and PUL constitutions. Two different phylogenetic trees were obtained using 9018 genome‐wide SNPs (*p* < 0.05) in the GWAS results (Figure 4a) and the combinations of 23 SNPs that were selected as the liver and lung phenotype‐specific SNPs in HEP and PUL constitutions and genome‐wide SNPs with *p* < 5 × 10^−3^ (Figure 4b). The number of clusters in these phylogenetic trees was different; compared to the phylogenetic tree that did not consider functional organ specificity (Figure 4a), the tree constructed with the liver and the lung phenotype‐related SNPs showed a clear difference between HEP and PUL (Figure 4b).

## 4 DISCUSSION

The eight constitutions disclose a wide spectrum of phenotypes, including the pulse shape, which is the major phenotypic determinant of the constitutions, and susceptibility to a certain disease or various health conditions as well as food choice and preference (Cho et al., 2013; Kim, Jang, et al., 2020; Kim, Kuon, et al., 2020; Kuon, 1965; Kuon, 1974; Kuon, 2003; Kuon & Berhardt, 2011). SNPs, the most common type of human variation, have been extensively investigated to determine the human traits and disease susceptibility. Many GWAS studies have uncovered novel links between SNPs and disease susceptibility (Dehghan, 2018; Marth et al., 2001; McCarthy et al., 2008). Therefore, we conducted a genome‐wide SNP study to verify the genetic makeup of eight inherited constitutions by selecting two opposing ECM types, HEP and PUL. To the best of our knowledge, this is the first genetic investigation to explore the genetic background underlying inherited ECM phenotypes.

In the present study, our GWAS analysis could not find specific SNPs that can differentiate between HEP and PUL, with the threshold for significance in GWAS analyses of common variants set at *p* < 5 × 10^−8^ (Fadista et al., 2016). On the other hand, most of the associations in our study seemed to have a moderate association or tendency. In fact, it is difficult to obtain concrete results because one SNP can affect multiple diseases or conditions, while a health condition or disease can be associated with multiple SNPs (Sivakumaran et al., 2011). Although there were neither genome‐wide significant SNPs (*p* < 5 × 10^−8^) nor suggestive SNPs (*p* < 1 × 10^−5^), Table 1 shows the SNPs associated with HEP and PUL constitutions at *p* < 1 × 10^−3^. This may be due to the small sample size that may lower the statistical power, which is one of the limitations of this study. The number of subjects (*n* = 63) was relatively small in the GWAS study. We believe that some of the moderate signals would become genome‐wide significant associations when the sample size is increased.

Among the SNPs listed in Table 1, only one SNP, rs1432364, appeared in the publication, suggesting its association with blood pressure response to methylphenidate treatment of attention‐deficit/hyperactivity disorder in children (Mick et al., 2011). This can be partially explained as HEP constitution is correlated with a high risk of metabolic syndrome (Kim, Jang, et al., 2020) and is known for the tendency of high blood pressure even in a healthy state (Kuon, 2003). When we searched for the top 100 SNPs associated with HEP and PUL constitutions by *p*‐value (data not shown), several SNPs that may be associated with either liver or lung traits were found based on previous SNP studies. Some studies have reported associations between rs329120 and rs329122 and body mass index (Akiyama et al., 2017; Pulit et al., 2019); rs329122 and type 2 diabetes mellitus (Mahajan et al., 2018; Spracklen et al., 2020; Zhao et al., 2017); rs2499604 and histologic features of nonalcoholic fatty liver disease (Chalasani et al., 2010); and rs11724031 and gut microbiota (Wang et al., 2016). Collectively, the characteristics of these SNPs may be consistent with the high risk of metabolic syndrome in HEP (Kim, Jang, et al., 2020). A study reporting the associations between rs11252717 and depression (Howard et al., 2017) may provide clues regarding the genetic influence on psychiatric characteristics in PUL subjects. Studies reporting the association between rs4712653 and neuroblastoma (Chang et al., 2017; Wang et al., 2011) and that between rs6556416 and neurological blood protein (Hillary et al., 2019) may also suggest the extension of neurological characteristics or possible vulnerability of PUL to neurological disease.

The ECM defines the HEP constitution as having strong liver and weak lung functions, while PUL constitution is the inverse (Kim, Jang, et al., 2020; Kuon, 1965; Kuon, 2003; Kuon & Berhardt, 2011). Thus, we investigated SNPs associated with either liver or lung function, as reported in the GWAS catalog (Buniello et al., 2019). Among the GWAS results of our subjects, eight SNPs were selected for their association with liver function; rs222054 and rs12743824 were reported to have significant genetic associations with adolescent NAFLD, the expression of the gene containing rs222054 was supposed to influence the vitamin D carrier protein, and rs12743824 was near the gene of lipid phosphate phosphatase related protein (Adams et al., 2013). Moreover, rs2499604 was found to be associated with serum alanine aminotransferase levels in patients with NAFLD (Chalasani et al., 2010). Further, rs4949718, rs12145922, and rs13030978 have also been reported to be associated with plasma levels of liver enzymes, such as alanine transaminase and aspartate transaminase (Chambers et al., 2011; Park et al., 2013). SNPs were chosen for their associations with lung function; rs10436951 and rs10113175 were reported to be associated with lung cancer susceptibility in smokers and non‐smokers, respectively, and rs2608029 is associated with lung adenocarcinoma, a subtypes of lung cancer (McKay et al., 2017). The other genetic variants, rs3009947, rs2348418, rs2637254, rs2571445, rs12201912, rs764129, rs10824305, rs2579762, rs541601, and rs755249 were reported to be associated with lung function such as forced expired volume in 1 second (FEV1), forced vital capacity (FVC) or FEV1/FVC, and chronic obstructive pulmonary disease (Kichaev et al., 2019; Shrine et al., 2019; Wain et al., 2017). In fact, it is difficult to obtain concrete results that presents specific SNPs reflecting specific feature because one SNP can affect multiple diseases or conditions, while a health condition or disease can be associated with multiple SNPs (Fadista et al., 2016). So the combination of SNPs can be considered as potential markers to differentiate two opposite constitutions HEP and PUL.

These SNPs related with liver and lung functions in HEP and PUL healthy subjects were selected and utilized to generate the phylogenetic tree of HEP and PUL using the SNPhylo program. Phylogenetic analysis and trees have been used for genetic and evolutionary studies in various organisms. Constructing more sophisticated phylogenetic trees became available based on large SNPs datasets due to advanced sequencing technology; however, it is also increasingly important to select suitable markers and use more proper algorithms or pipelines to produce trees which are more reliable and visually intuitive to understand the inter‐relationship among biological entities (Basibuyuk et al., 2015; Hennig, 1966). As a result of phylogenetic analysis in this study, a distinct difference or cluster emerged between the two constitutions (Figure 4b). It was confirmed that those functionally selected SNPs could be suitable markers and SNPhylo helped in understanding or interpreting the difference of HEP and PUL group easily showing the visually intuitive phylogenic tree.

In fact, the phylogenetic tree may represent evolutionary relationships among organisms, even though it does not provide conclusive evidence (Lee et al., 2014). However, here we focused on exploring a possible genetic difference between two human group classified by the eight constitutions with phylogenetic analysis. The distinctively divided two clusters reflect the two genetically distinguishable constitutional groups of HEP and PUL, suggesting that the combination of these 23 SNPs associated with the function of each strongest organ, liver in HEP and lung in PUL can be utilized to genetically distinguish HEP and PUL constitutions. To date, the evidence of genetic background in ECM has been reported, and the representative diagnostic method has mainly been based on phenotypes such as pulse shape (Kuon & Berhardt, 2011; Shin et al., 2009). The present study can support the hypothesis that the eight constitution phenotypes are inherited and based on genetics and that genetic markers may exert a potential role as objective and accurate diagnostic assessment of the eight constitutions.

Moreover, once phenotypically classified and now phylogenetically distinguishable constitutions in human may deepen our understanding of approach to human health and disease. In the same species, human, healthy people can react differently to the same external stimulus. For example, under the same temperature one can sweat a lot while the other rarely sweats. After sweating, one can may feel tired whereas the other may feel refreshed. According to ECM, people in HEP constitution are considered to be healthy when they sweat well while people in PUL constitution are recommended to avoid sweating for heathy condition (Kuon, 2003). After taking the same food such as meat, one can digest fast and easily while the other feel rather discomfort. According to ECM, people in HEP constitution are recommended to eat meat while people in PUL constitution are not (Kuon, 2003). It was reported that the amount of calorie or fat intake was different in HEP and PUL, serum lipid profiles were significantly higher in HEP than in PUL (Cho et al., 2013) and that nutrition intake based on the food regimes of ECM might influence on quality of life (Kim, Kuon, et al., 2020). Finding the genetic evidence underlying these differences in human, the eight constitutions may provide more information on physiological or pathological conditions and responses according to constitutions and a clue on human evolution in the way of thinking that evolutionary change might occur after accumulating a difference of physiological response tendency to external stimulus.

Individuals with the same constitution can have different genotype distributions of genes that are not involved in each constitution, which may cause different phenotypes in the same constitution, especially because many genes are involved in individual health conditions and diseases. This kind of genetic difference can be a confounding factor in determining the constitution by phenotype only, even though different health conditions and disorders do not affect the constitution type theoretically. Thus, combining the genotypes specific to each constitution may increase the power to classify the constitution types.

In this first trial to investigate the genetic background of ECM, we found a clue that may support the presence of a genetic mechanism behind the peculiar phenotypes of the eight constitutions. The skewed number of subjects was mentioned above as one of limitations in our study. Another limitation of the study would be lack of the diagnostic data from other diagnostic tools or lack of cross‐validation with more than one ECM expert, as the diagnosis of the eight constitution was carried out only by one ECM specialist. The facts that the skillful ECM expert who specializes and has been experienced in ECM for about 20 years participated and that the diagnosis was carried out more than twice with time differences in the present study might supplement this limitation though. In the previous studies, clinical experience of the practitioner was reported to be critical for the consistency and accuracy of ECM pulse diagnosis (Shin et al., 2009) and other diagnostic tools such as questionnaire was developed to secure objectivity but also to make up for lack of clinical experience of practitioners in ECM (Lee et al., 2012). The other limitation is the different sex distribution between the HEP and PUL groups. In the present study, the same number of men and women was included in the HEP group but not in the PUL group, showing statistically significant difference of sex distribution between the groups (data not shown). We investigated if there are any associations between sex and those top 100 SNPs for the significant index of the HEP and PUL constitutions or those 23 SNPs, which were selected as being liver‐ and lung‐phenotype‐specific and found to show a clear difference between HEP and PUL in phylogenetic analysis, by looking through the GWAS catalog (Buniello et al., 2019). As a result, we could not find any SNPs which have associations with sex, have a role in sex hormone, or are on the X or Y chromosome. ECM presupposes that the constitution classifying the individual's unique pathophysiological and psychological characteristics into eight categories is an inherited nature and independent from sex or age and there were more females than males in the subjects of the other ECM studies (Kim, Jang, et al., 2020; Kim, Kuon, et al., 2020). However, we expect the future study would investigate if those SNPs would also be reported in the female‐ or male‐only group in larger HEP and PUL samples and if other SNPs related to other eight constitutions besides HEP and PUL would have no associations with sex or sex hormone.

For further studies, it is recommended to reproduce the same conditions by enrolling both healthy and unhealthy volunteers with HEP or PUL constitutions with a larger number of subjects to reach statistical significance thresholds. More than two diagnostic tools or ECM experts for cross‐validation should be required to diagnose the eight constitutions for qualitative data. To clarify the premise of ECM about sex and age which do not affect the eight constitutions, the sex distribution in the eight constitution groups should be considered during the recruitment. Further, our study can be extended to all eight constitution types to find SNPs that may determine each constitution and major organ functions. The analysis of each SNP function could also help to understand the variable susceptibility to certain disease conditions in each constitution, as well as dietary guidelines and lifestyle modifications that can fit to their constitution. The association of the effectiveness of constitutional acupuncture or food regimens as therapeutic and preventative approaches could be examined further based on newly identified genetic makeups. We hope that our study results can prompt future genetic studies to clarify the genetic variations in ECM and provide clues as to the personalized approach determined by an individual's own inherited constitution types. We also expect that this genetic study can be a steppingstone to apply ECM to other ethnic groups beyond human variation.

## AUTHOR CONTRIBUTION

C.K., K‐W.H., S.O., K.K., S‐W.C., and H.J. contributed to the conception of the study and study design; K‐W.H. and D‐H.P. to the microarray and GWAS; S.C. and Y.P. to the patient samples and data collection; K‐W.H., D‐H.P., S.C., S.O., K.K., Y.P., and H.J. to the data analysis and interpretation; H.J. to the article draft; and C.K., K‐W.H, H.J., and S‐W.C. to the writing and editing; All authors have reviewed the manuscript.

## FUNDING INFORMATION

N/A.

## CONFLICT OF INTEREST

The authors declare no conflicts of interest. The corresponding author is responsible for submitting a competing interest statement on behalf of all authors of the paper.

## Supporting information


Table S1
