# Supplementary material for: Lung‐ and liver‐dominant phenotypes of Korean eight constitution medicine have different profiles of genotype associated with each organ function
Source: Physiol Rep. 2022 Sep 6;10(17):e15459. doi: 10.14814/phy2.15459 (PMC9446411; doi:10.14814/phy2.15459)
Supplement: Supplementary file 1 — Table S1 [file PHY2-10-e15459-s001.docx]

**Supplementary**

Table. Age and sex distributions of participants in HEP and PUL groups

| Constitutions | HEP (%) | | PUL (%) | |  |
| --- | --- | --- | --- | --- | --- |
| Age groups | Male | Female | Male | Female | Total |
| 20-29 | 0 | 0 | 0 | 3 | 3 |
| 30-39 | 3 | 3 | 1 | 2 | 9 |
| 40-49 | 3 | 3 | 1 | 8 | 15 |
| 50-59 | 4 | 2 | 0 | 6 | 12 |
| 60-69 | 3 | 4 | 0 | 6 | 13 |
| 70-79 | 2 | 2 | 1 | 2 | 7 |
| 80< | 1 | 2 | 0 | 1 | 4 |
| Total | 16 | 16 | 3 | 28 | 63 |
